# Bimekizumab for refractory hidradenitis suppurativa and associated inflammatory arthropathies

**DOI:** 10.1016/j.jdcr.2026.03.025

**Published:** 2026-03-19

**Authors:** Christina Tolete, Reina Tolete, Adam Friedman, Leonardo Tjahjono

**Affiliations:** a Department of Dermatology, George Washington University, School of Medicine and Health Sciences, Washington, District of Columbia; b The University of Santo Tomas Faculty of Medicine and Surgery, Manila, Philippines

**Keywords:** ankylosing spondylitis, arthropathies, bimekizumab, hidradenitis suppurativa, interleukin-17, psoriatic arthritis

## Introduction

Hidradenitis suppurativa (HS) is a chronic, relapsing inflammatory disease characterized by painful nodules, draining sinus tracts, and scarring, most often affecting the axillae, groin, and inframammary regions.^1^^,^^2^ HS frequently coexists with systemic inflammatory disorders, including Crohn’s disease and inflammatory arthropathies such as psoriatic arthritis (PsA) and ankylosing spondylitis (SpA).^3^^,^^4^

Management of HS is challenging and coexistence of comorbidities further lower patients’ quality of life. Therapeutic goals include reducing pain, preventing new lesion formation, and limiting disease progression.^5^ Adequate control often requires multimodal approach and regimen, and it can overwhelm patients. Therefore, simplifying regimen to treat the comorbidities adequately may increase adherence and lower potential adverse events. We describe 4 patients with concomitant recalcitrant HS and recalcitrant inflammatory arthropathy (IA) who improved while on bimekizumab after inadequate response or intolerance to multiple combinations systemic and biologic therapies.

## Results

The cohort comprised 4 patients (3 females and 1 male, ages 34-52) with concomitant HS and IA, including PsA and spondyloarthritis (SpA). All had severe HS with International Hidradenitis Suppurativa Severity Score System (IHS4) score of at least 14, refractory to at least 2 biologic classes, most commonly TNF-α and IL-17A inhibitors, with some having also failed JAK or IL-23 inhibitors. Prior to initiation of bimekizumab, patients only had a change in activity scores of their respective diagnoses of 10% at most despite trying combinations of treatments of at least 4 months. We used approved dosing of subcutaneous 320 mg bimekizumab loading dose of every 2 weeks for the first 16 weeks, and then every 4 weeks thereafter (Table I). We used the IHS4, the Ankylosing Spondylitis Disease Activity Score (ASDAS), and the Disease Activity index for Psoriatic Arthritis (DAPSA) because these validated continuous indices more accurately capture longitudinal disease activity over time than categorical response measures such as the American College of Rheumatology response criteria or Hidradenitis Suppurativa Clinical Response, and they represent the standard disease activity metrics employed in our combined dermatology-rheumatology referral clinic.

**Table I:** Summary of 4 patients with concomitant hidradenitis suppurativa and inflammatory arthropathy treated with bimekizumab

Gender	Age	Hidradenitis suppurativa initial IHS4 prior to bimekizumab	Comorbid inflammatory arthropathy	Inflammatory arthropathy score (DAPSA for PSA, ASDAS for SpA) prior to bimekizumab	Prior treatments	HS and IA status 8 wk after bimekizumab	HS and IA status 16 wk after bimekizumab	Notes
Female	43	25	SpA	2.9	Ada, Sec, Mtx, SCS	12, 1.7	4, 1.5	MTX and SCS were combined with either adalimumab or secukinumab prior to bimekizumab initiation. Bimekizumab therapy led to complete resolution of sacroiliitis and bone marrow edema.
Female	34	22	PsA	27	Ada, Ixe, Sec, Mtx, Upa, Ifx	14, 17	6, 6	Concomitant acne conglobata improved, with reduction in swollen and tender peripheral joints. MRI demonstrated decreased tenosynovitis and enthesitis.
Female	52	17	PsA	30	Ada, Ixe, Rzk, Gsk, Ifx, Sec	10, 14	2, 3	Concomitant severe cutaneous psoriasis resolved, with MRI showing reduction in tenosynovitis and enthesitis.
Male	45	14	SpA	3.8	Ada, Eta, Mtx, Sec, Ixe	9, 1.6	5, 0.9	HLA-B27 positive. MRI with complete resolution of sacroiliitis and bone marrow edema.

*Ada*, Adalimumab; *BASDAI*, Bath Ankylosing Spondylitis Disease Activity Index; *CR*, complete response; *Eta*, etanercept; *Gsk*, guselkumab; *HLA*, human leukocyte antigen; *Ifx*, infliximab; *Ixe*, ixekizumab; *MRI*, magnetic resonance imaging; *Mtx*, methotrexate; *NR*, no response; *PR*, partial response; *PsA*, psoriatic arthritis; *Rzk*, risankizumab; *SCS*, systemic corticosteroids; *Sec*, secukinumab; *SpA*, spondyloarthritis; *Upa*, upadacitinib.

**Fig 1:**
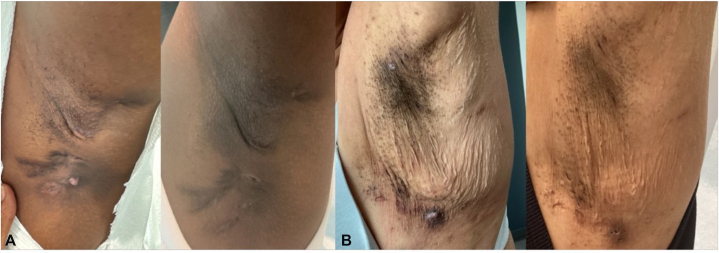
Clinical improvement of axillary hidradenitis suppurativa following bimekizumab. **A,** Patient 1: baseline (*left*) showing active nodules and inflammation; follow-up (*right*) demonstrating marked reduction in lesion count and decreased induration with residual post-inflammatory changes. **B,** Patient 3: baseline (*left*) demonstrating active inflammatory nodules, draining tunnels and indurated plaques; follow-up (*right*) showing resolution of active lesions with decreased erythema, flattening of nodules, cessation of draining tunnels, and residual postinflammatory hyperpigmentation and scarring.

A 43-year-old woman with SpA and HS refractory to a combination of 40 mg weekly adalimumab (and eventually this was switched 300 mg subcutaneous secukinumab every 2 weeks), 15 mg weekly methotrexate, and 15 mg of daily prednisone was tried for 6 months without any improvement in ASDAS and IHS4 score. The patient’s IHS4 score decreased by 21 points after 16 weeks of bimekizumab (Fig 1, *A*), showing complete resolution of sacroiliitis and bone marrow edema on magnetic resonance imaging and a decrease of ASDAS score from 2.9 to 1.5 after 16 weeks. A 34-year-old woman with HS score of IHS4 22 and PsA score of DAPSA 27 demonstrated improvement with IHS4 and DAPSA decreasing to 6 and 6, respectively, after 16 weeks of bimekizumab despite being previously refractory to multiple sequential biologics (40 mg weekly adalimumab, 80 mg weekly ixekizumab, 300 mg secukinumab every 2 weeks, 30 mg daily upadacitinib, 10 mg/kg infliximab every 4 weeks), with concurrent improvement in acne conglobata, reduction in tender swollen peripheral joints and decreased tendosynovitis and enthesitis on magnetic resonance imaging. A 52-year-old woman with concurrent PsA and severe psoriasis also had a decrease in IHS4 by 15 points (Fig 1, *B*) and DAPSA score by 27 points following bimekizumab after inadequate response to 6 prior sequential biologics (40 mg weekly adalimumab, 80 mg weekly ixekizumab, 150 mg risankizumab every 3 months, 100 mg guselkumab every 2 months, 10 mg/kg infliximab every 4 weeks, and 300 mg secukinumab every 2 weeks), with magnetic resonance imaging evidence of reduced tendosynovitis and enthesitis. Finally, a 45-year-old HLA-B27 – positive man with SpA and HS nonresponsive to 4 prior sequential biologics, 40 mg weekly adalimumab, 50 mg weekly etanerecept, 300 mg secukinumab every 2 weeks, and 80 mg ixekizumab every 4 weeks, improved his IHS4 score with a 9-point-decrease and ASDAS score with 2.9-point-decrease, with complete resolution of sacroiliitis and bone marrow edema (Table I).

Overall, bimekizumab demonstrated consistent dual improvement across cutaneous and musculoskeletal domains in patients with refractory HS and IA, including those previously unresponsive to multiple biologic mechanisms. Additionally, all patients tolerated the bimekizumab treatment throughout with no adverse events reported, including absence of oral candidiasis. Complete blood count with differential and comprehensive metabolic panel showed no abnormalities or any changed during 8th and 16th week follow up.

## Discussion

Managing HS presents significant clinical challenges due to its chronic, relapsing course and its frequent overlap with systemic inflammatory conditions such as PsA and SpA. Standard treatment regimens often involve combinations of antibiotics, hormonal agents, TNF-α inhibitors, and IL-17 antagonists, yet many patients remain refractory requiring multiple agents with overlapping mechanisms and cumulative side effects. While clinical trial endpoints for PsA (ACR20) and axial spondyloarthritis (ASAS40) represent only 20% and 40% improvement thresholds, respectively, these remain modest compared to PASI100 in psoriasis or EASI75-90 in atopic dermatitis, highlighting a clear unmet need for agents capable of achieving deeper and faster responses across inflammatory spectra. Our findings highlight that bimekizumab not only improved cutaneous HS manifestations but also significantly ameliorated the concomitant IA in all 4 patients, thus demonstrating true cross-domain efficacy. This dual benefit underscores its therapeutic potential in simplifying management for patients burdened by both skin and joint disease. Bimekizumab is currently approved for several immune-mediated inflammatory diseases, including plaque psoriasis, PsA, axial spondyloarthritis, SpA, and more recently, HS.

Patients in this series demonstrated meaningful clinical improvement with bimekizumab despite prior inadequate response to selective IL-17A inhibitors such as secukinumab and ixekizumab. Bimekizumab uniquely inhibits both IL-17A and IL-17F, cytokines with overlapping but nonredundant proinflammatory effects. Emerging data suggest that IL-17F is abundantly expressed in HS lesions, PsA synovium, and SpA enthesitis, which may explain bimekizumab’s efficacy despite refractory to other biologics in our cohorts.^6^, ^7^, ^8^, ^9^

The concept of a single biologic providing benefit across both HS and IA is not unprecedented. TNF-α inhibitors, particularly adalimumab, are commonly used in patients with comorbid HS and inflammatory arthritis, with real-world studies and case reports supporting their dual efficacy.^10^^,^^11^ However, many patients experience inadequate response or loss of efficacy with TNF inhibition, especially in severe or refractory HS.^12^ Notably, patients with prior TNF-α inhibitor failure often demonstrate attenuated responses to subsequent IL-17A or IL-12/23 inhibitors.^13^^,^^14^ In this context, the consistent improvement observed with bimekizumab in patients who were previously refractory to TNF-α and IL-17A-selective therapies suggests that dual IL-17A/F inhibition may represent an effective alternative strategy for managing overlapping cutaneous and musculoskeletal disease. Our patients, many of whom were recalcitrant to several biologic classes, experienced rapid and meaningful improvement by week 16, surpassing the results from the pivotal phase 3 trials, as all of our patients achieved Hidradenitis Suppurativa Clinical Response 75 and significant IHS4 score improvement better than that of the trial.^13^^,^^15^ Additionally, our cohort’s DAPSA score improvement for PsA is consistent with findings from pivotal phase 3 BE OPTIMAL trials. In that study of biologic DMARD-naive patients with SpA, 44% of patients treated with bimekizumab achieved an ACR50 response at week 16 compared with 10% receiving placebo and 30% receiving adalimumab. Although dosing differed in our cohort, these results support the joint improvements observed in our patients.^16^ The DAPSA score improvements in the PsA cohort underwent meaningful change and improved from severely high disease activity to remission and low disease activity.^17^^,^^18^ This real-world speed and magnitude of response suggest bimekizumab may outperform RCT benchmarks in highly treatment-resistant populations.

Equally important, bimekizumab allowed consolidation of multiple systemic therapies into a single biologic, improving adherence, minimizing medication burden, and enhancing overall quality of life. For patients previously dependent on complex multidrug regimens (eg, TNF-α inhibitors, IL-17A inhibitors, JAK inhibitors, and corticosteroids), transitioning to 1 agent with broad immunologic coverage represents a major step toward streamlined, patient-centered care.

Even in patients with overlapping, recalcitrant immune conditions such as severe psoriasis, PsA, or SpA, bimekizumab demonstrated durable improvement across all domains with a single therapeutic mechanism, emphasizing its unique ability to address systemic inflammation comprehensively.

Taken together, these results underscore the rationale for this report, highlighting bimekizumab’s distinct efficacy, rapid real-world improvement, and its ability to simplify complex treatment regimens in patients with overlapping inflammatory diseases, ultimately providing an integrated and impactful therapeutic option for those unresponsive to prior biologic therapies.

## Conflicts of interest

Dr Tjahjono has served as a consultant and/or speaker for Arcutis, Bristol Myers Squibb, Eli Lilly, Incyte, Leo pharma, and Galderma. Dr Friedman was on the consulting/ad board of La Roche Posay, Galderma, Kenvue, Microcures, Leo Pharma, Pfizer, Hoth Therapeutics, Zylo Therapeutics, Mino Labs, J&J, Arcutis, Lilly, UCB, Novartis, UCB, Regeneron/Sanofi, Takeda; was a speaker for Regeneron/Sanofi, J&J, Incyte, UCB, Galderma, Arcutis, Lilly, Pfizer, Novartis; received grants from Pfizer, Lilly, Galderma, Incyte, J&J, Abbvie, Loreal, and Regeneron/Sanofi. Dr Tolete and Miss Christina Tolete do not have any disclosures.
